# The “Neurospeed” game: a fun tool to learn the neurological semiology

**DOI:** 10.1186/s12909-022-03316-8

**Published:** 2022-03-31

**Authors:** Sinead Zeidan, Solenne Baltaze, Béatrice Garcin, Astrid de Liège, Jennifer Doridam, Laure Josse, Bertrand Degos

**Affiliations:** 1 Neurology Department, APHP, Hôpital Avicenne, Hôpitaux Universitaires de Paris - Seine Saint Denis, Sorbonne Paris Nord, Bobigny, France; 2 Medicine Department, UFR SMBH, Sorbonne Paris Nord, Bobigny, France; 3 Healthcare Simulation Center, UFR SMBH, Sorbonne Paris Nord, Bobigny, France; 4 Center for Interdisciplinary Research in Biology, Collège de France, CNRS UMR7241/INSERM U1050, Université PSL, Paris, France

**Keywords:** Medical education, Neurological semiology, Gamified learning

## Abstract

**Background:**

Neurological semiology is often considered by medical students as particularly difficult to learn. Finding alternative teaching methods may improve students’ motivation and understanding of this field.

**Methods:**

We developed the “Neurospeed”, a game to learn neurological syndromes. We assessed its efficiency on short-term learning of neurological syndromes in third-year medical students, through Multiple Choice Questions (MCQs) before and after the game session. Students’ satisfaction was evaluated by a satisfaction survey.

**Results:**

Out of the 199 third-year medical students of the Faculty of Medicine Sorbonne Paris Nord, 180 attended the Neurospeed in December 2020, and 148 answered 20 Multiple Choice Questions before and after the game, with significant improvement of their score (*p* < 0.001). Most of the participants agreed that the game was playful, stimulating, and helpful to learn neurological semiology.

**Conclusions:**

Overall, our results show that the Neurospeed game is an interesting tool as a complement to traditional lectures. Further studies are necessary to compare the efficacy of different types of serious games on short-term and long-term learning of neurological semiology.

**Supplementary Information:**

The online version contains supplementary material available at 10.1186/s12909-022-03316-8.

## Background

Neurological semiology encompasses many elements for students to learn [1], and clinical training generally does not allow for the full range of neurological syndromes to be seen at the patient’s bedside in the first years of medical school. Neurological semiology is often considered by medical students to be one of the most difficult clinical areas to learn [2, 3], resulting in neurophobia [4–7]. In our university, as in many others, a large part of medical semiology is taught in lectures. However, lectures are teacher-centered and associated with limited levels of engagement for the learner [8]. Therefore, it is essential to find alternative ways to teach neurological semiology to improve student motivation and engagement, and to provide a better understanding of this broad field.

Active learning is a growing trend in universities, and has been associated with better outcomes than traditional lectures among undergraduate science, engineering and mathematics students [9]. The learner is encouraged to actively use knowledge rather than passively learn [10], as illustrated in Miller’s pyramid for medical education [11]. Therefore, active learning can be helpful, taking different forms, such as problem-based learning or flipped-classroom formats [8, 12], with positive effects on learning as shown in a 2018 meta-analysis [13].

Game-based learning is gaining interest [14, 15] as it is active, student-centered, and rewarding. It can involve multiple sensory inputs and can be repeated as often as needed. Various adaptations of existing games have been proposed [16, 17], as well as online projects [18], which are particularly interesting in the context of lockdowns due to the COVID-19 pandemic [19].

As an alternative or in addition to the Neurological Hat Game previously developed by Garcin et al. [17], we developed the “Neurospeed”, a new game to learn the semiology of neurological syndromes. We evaluated its effectiveness on short-term learning through Multiple Choice Questions (MCQs) and assessed students’ satisfaction through a satisfaction survey.

## Methods

### Design

This observational prospective study was conducted at the Faculty of Medicine Sorbonne Paris Nord in December 2020. It was approved by the local ethics committee (Comité Local d’Ethique pour la Recherche Clinique des Hôpitaux Universtaires Paris Seine Saint-Denis Avicenne-Jean Verdier-René Muret, CLEA-2020-157), and the internal review board of the Faculty of Medicine Sorbonne Paris Nord, which authorized the inclusion of the Neurospeed game in the medical curriculum of third-year medical students.

### Participants

All third-year medical students were included, as the Neurospeed game was integrated into their neurology curriculum. An online interactive board was generated to record all responses of the third-year medical students participating in this teaching. Students completed questionnaires on electronic tablets, before and after the Neurospeed session. The teaching took place during a one-week revision period of the neurological semiology. Groups of 6-8 students were made among the students. Each group was supervised by a neurologist (BD, JD, AdL). All participants gave their oral informed consent.

### The Neurospeed game

A deck of 78 cards was designed by BD, with a neurological sign or symptom written on each card (see Additional file 1). Two senior neurologists (BG and JD) reviewed all words and verified that each card had a correct and properly spelled semiological term. Some cards could be used for different syndromes: for example, “dysarthria” and “tremor” could be part of a cerebellar syndrome, and part of a parkinsonian syndrome; “motor weakness” could be seen in a pyramidal syndrome and in a peripheral neuropathy. There were some cards with distractors, which did not belong to any syndrome, such as “dystonia” and “ballism”.

Each game session involves 6-8 participants and proceeds as follows: the cards are distributed, face down, to the players arranged in a circle. For the first round, all players turn over the top card of their deck at the same time and for subsequent rounds, players take turns adding a card. At any time, if at least three of the cards turned over show a sign or symptom that constitutes a neurological syndrome, then the players should hit the table with their palms as quickly as possible. The player who hits the table first must say which syndrome is evoked and why. If that player is wrong, they get the cards back. If they are right, the last player who hits the table gets the returned cards. In addition, the player who reacts first and finds a syndrome can discard one of their cards by giving it to one of the players. The first player to run out of cards wins.

During and after each game session, participants could discuss their difficulties or questions, and the neurologist supervising the session provided explanations. The whole session lasted approximately 60 min, including two card-playing sessions and the debriefing session.

### Multiple Choice Questions (MCQs)

The benefit of the Neurospeed game on short-term learning was assessed through 20 Multiple Choice Questions (MCQs) on neurological semiology (see Additional file 2). This questionnaire was designed by BD and corrected by BG to assess knowledge about symptoms and diagnoses. Just before starting the Neurospeed game, participants had to answer the 20 MCQs, which they had never seen before. They did not get their scores or any feedback on correct answers afterwards, to avoid interfering with the Neurospeed game. Then, after completion of the Neurospeed session, the students were assessed with the same 20 MCQs, but proposed in a different order. The delay between the pre-test and post-test MCQs was around 3 h. Only fully correct MCQs were counted as valid (1 point per MCQ), and students were given a limited time of 15 min to complete the MCQs. Possible knowledge scores ranged from 0 to 20.

### Satisfaction survey

Participants completed a satisfaction survey immediately after the second MCQs session. The satisfaction survey consisted of 8 questions (see Additional file 3) that students were asked to answer according to a 5-choice Likert scale (1. Strongly agree, 2. Agree, 3. Neutral, 4. Disagree, 5. Strongly disagree). It has been previously used in similar studies [17, 20].

### Grades on the neurology exam

In 2018 and 2019, the semiology curriculum of third-year medical students included a neurological version of the Hat Game [17]. The grades on the neurology exam of the third-year medical students who attended the Neurospeed game were compared to those from the two previous years (2018 and 2019) who attended the neurological Hat Game.

### Statistical analysis

Statistical analyses were conducted using R software version 4.0.4., with R studio version 1.4.1106. Categorial variables were described as counts and percentages, and quantitative variables as means and standard deviations (normal distribution). T-tests or paired t-tests were used to compare quantitative variables. Associations between categorial variables were assessed using the Chi-square test.

## Results

### Students

**Fig. 1:**
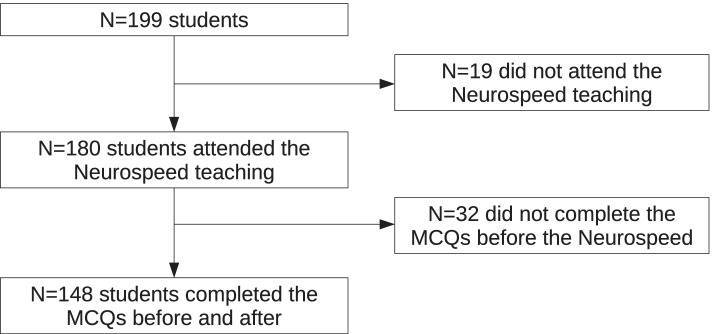
Flowchart of the Neurospeed study. MCQs = Multiple Choice Questions

Out of the 199 third-year medical students, 148 took part in the Neurospeed teaching and answered the Multiple Choice Questions (MCQs) before and after the Neurospeed teaching. Nineteen students did not attend the Neurospeed teaching, and 32 did not answer the MCQs before the Neurospeed teaching because they were late (Fig. 1). The majority of participants were female (*N* = 92/148, 62.2%) and the mean age was 21.1 ± 2.1 years.

### MCQs results

**Fig. 2:**
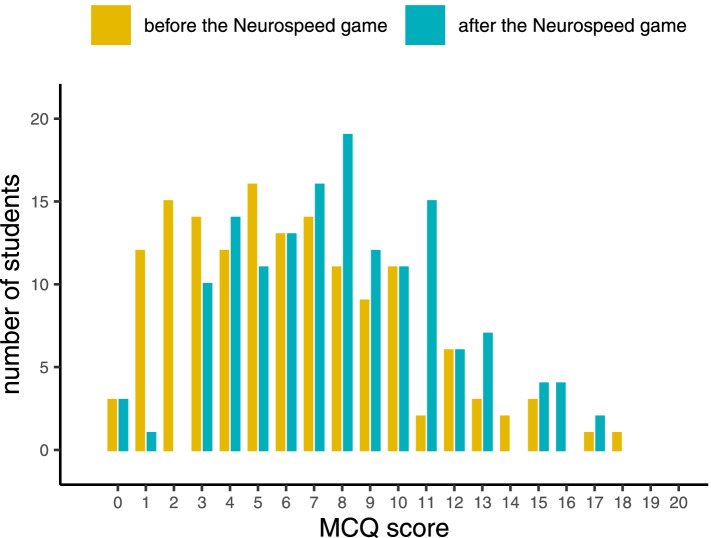
Distribution of students’ MCQ scores before and after the Neurospeed teaching

Before the Neurospeed teaching, the mean MCQ score was 6.13 ± 3.87. The mean score after the Neurospeed teaching was 8.03 ± 3.65 (*p* = 1.28 × 10^− 15^), with a mean of the differences of 1.89 (Fig. 2). After the game, 49 out of 148 (33.1%) students had scores greater than or equal to 10, versus 29 students (19.6%) before the game (*p* = 2.36 × 10^− 10^).

### Satisfaction survey

Among the 148 students who attended the Neurospeed teaching and answered the MCQs, 123 (83.1%) completed the satisfaction survey after the game, yielding a total of 1107 answers. The results are presented in Table 1 and show that the students were overall satisfied with the Neurospeed.

**Table 1:** Assessment of students’ satisfaction survey regarding the Neurospeed (*N* = 123)

Proposals about the Neurospeed	Mean ± SD^a^
It is playful	1.22 (± 0.47)
It is a stimulating game	1.33 (± 0.61)
It helped better understand neurological semiology	1.40 (± 0.67)
It helped better remember neurological semiology	1.47 (± 0.69)
It was useful for reviewing the upcoming exam	1.41 (± 0.60)
It increased motivation to learn neurological semiology	1.37 (± 0.65)
Terms used were appropriate	1.71 (± 0.86)
It should be repeated in the future	1.33 (± 0.69)
It should be extended to other medical specialties	1.33 (± 0.72)

^a^from strongly agree (1) to strongly disagree (5)

### Grades on the neurology exam

**Fig. 3:**
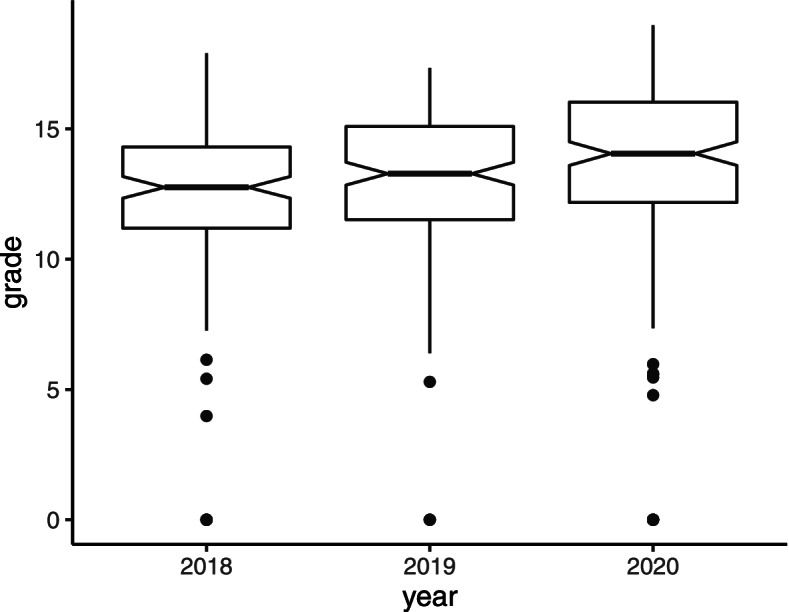
Grades on the Neurology Exams for the years 2018, 2019 and 2020 (exclusion of the students who did not participate in the neurological Hat Game or the Neurospeed game)

The grades of the 180 third-year medical students who participated in the Neurospeed game were compared to those of third-year medical students from the two previous years who participated in the Neurological Hat Game [14] (*n* = 167/177 in 2019, *n* = 150/169 in 2018). The mean score on the neurology exam was significantly higher for the year 2020 (year of the Neurospeed teaching) compared to year 2018 (mean 13.46 ± 3.88 vs. 12.23 ± 3.47, *p* = 0.003), but was not significantly different from year 2019 (mean 12.86 ± 2.95, *p* = 0.104) (Fig. 3). The percentage of students scoring 10 or higher (out of 20) was not significantly different from the two previous years: 88.9% (*n* = 160/180) in 2020, vs. 88.0% (*n* = 147/167, *p* = 0.801) in 2019, and 87.9% (*n* = 123/140, missing data: *n* = 10, *p* = 0.775) in 2018.

## Discussion

This study shows that the Neurospeed game improves short-term learning of neurological semiology, and is very appreciated by students. Indeed, students had better MCQ scores after the Neurospeed game, and most of them strongly agreed that it is a playful, useful and stimulating game.

This work has several limitations. First, the delay between the pre-test and post-test MCQs was short, resulting in a potential test/re-test effect, and preventing any study of the impact of the game on long-term memory. Unfortunately, the time available for each session was limited, and the organization was complicated by the ongoing pandemic. As a result, only short-term memory could be evaluated. A long-term assessment, and the use of different questions in the MCQs for the pre- and for the post-test, would help mitigate these limitations in future studies. In a recent study (Clément et al.), we demonstrated that the Hat Game, which is, like the Neurospeed, a card game aimed at learning the neurological semiology, maintained performance improvement 3 months after the game [20]. Second, since all students were enrolled in the Neurospeed game, we did not have a control group with a lecture for comparison. Third, the Neurospeed game focuses on memorization of neurological syndromes through repetition: one could argue that clinical reasoning is not involved. However, a neurologist supervised the session in order to correct mistakes and to provide precision regarding neurological semiology and physiology. The Neurospeed game, and gamified learning in general, cannot be a substitute for a teacher, and is intended as a means of teaching, in addition to traditional lectures. Fourth, as mentioned above, the organization of this teaching was complicated by the health situation related to COVID-19 pandemic, with each session being time-limited to rotate small groups of students, resulting in a loss of data due to late arrival or early departure of some students.

The Neurospeed game is convenient to use: it only requires a deck of cards and can be played anywhere. It is inexpensive, unlike simulation games that require sophisticated platforms, or computerized games. Different games can be played with the same material, since it is possible to play the Neurological Hat Game [17] or the Neurospeed with the same deck of cards. Furthermore, the game can be adapted to the level of the students by adding or removing certain cards.

Several aspects of the Neurospeed game may participate in learning improvement. First, it involves reading and verbal inputs: sensory read-write and auditory modalities are common among students, who generally prefer non-unimodal learning [21–23]. Second, it engages high levels of attention, which is important for working memory consolidation [24]. Third, the active participation, positive mood (as reflected by the students’ satisfaction survey), motivation and reward associated with the game have a positive effect on the learning experience [25–30], notably through the involvement of mesocorticolimbic pathways [31, 32]. Another interesting aspect of the Neurospeed is *interleaving*: students quickly switch between different types of neurological syndromes throughout the game, and this learning technique has been recognized as effective in medical education [33]. Regarding the conceptual framework of the Neurospeed teaching, several learning theories can be invoked. According to the cognitive load theory, which focuses on optimizing working memory, the Neurospeed can help novice medical students decrease their intrinsic load when performing the neurological examination of a patient, since it trains them to quickly recognize semiological patterns [34]. As developed in social cognitive learning theory, students can learn by observing and listening to each other’s explanations during the Neurospeed, and through enhancement of their self-efficacy by positive emotions generated by this playful, sane competition [35]. In this study, we compared the grades of the students to previous years, to assess the impact of the game on neurology exam results. The grades on the neurology exam were improved compared to year 2018, but not compared to year 2019. However, it is important to note that students who took the Neurology exam on year 2018 and 2019 had another type of game-based learning, the “Neurological Hat Game” [17]. In a future randomized controlled study, the Neurological Hat Game and the Neurospeed game could be compared to assess whether one game is more effective than the other. The combination of both games could also be tested to determine if it has an additive effect. The majority of students had MCQ scores below 10/20 even after the Neurospeed game. This is comparable to previous results obtained after the Neurological Hat Game [17] and is partly due to the scoring being on an “all-or-nothing” basis. However, this suggests that gaming alone is insufficient, and that several repetitions of the games may be necessary for a better memorization. Overall, our results tend to confirm those observed with the “Neurological Hat Game” by Garcin et al. [17].

Active and game-based learning have limitations: the sessions can be time-consuming, and in the medical field, with high academic requirements in a limited amount of time, it cannot be applied to the whole program and should probably be limited to some specific subspecialties [12]. In addition, a lack of buy-in from students may occur, for instance due to the increased preparation time, and limit the implementation of this type of teaching method [12, 36]. Another issue is the need for trained faculty staff, familiar with adult learning theories and game-based education [36]. Although gamified learning is promising, evidence is lacking regarding the effectiveness of educational games as a teaching strategy for medical students [37]. In a systematic review of serious games in medical education by Gorbanev et al., the effectiveness of these games was moderate, and the pedagogical strategies used were mostly behaviourist and cognitivist, focusing on memory and skill development through repetition [14]. In the field of neurology, several studies have been promising, and serious games have met the expectations of students, who often consider neurological semiology to be particularly difficult to grasp [5, 17, 38–40]. However, evidence supporting the use of game-based learning in neurology remains scarce [36]. Gamified learning formats could be useful for specific topics that particularly trigger neurophobia, such as neurological semiology [5, 41]. Table 2 summarizes five studies of gamified active learning in neurology, among medical students and residents. Overall, students were satisfied with these different types of interventions. Roze et al. showed positive results for long-term retention of neurological semiology, with a role-play training program [38]. Outcomes were assessed with written exams in most studies, including ours.

**Table 2:** Gamified active learning in Neurology for medical students/residents

Study	Lim et al., 2008 [40]	Roze et al., 2018 [38]	Garcin et al., 2019 [17]	Schuh et al., 2008 [42]	Raskurazhev et al., 2021 [16]
Location	National University of Singapore	Faculty of Medicine, Sorbonne University, Paris	Faculty of Medicine, Sorbonne University, Paris	Henry Ford Hospital, Detroit, USA	Research Center of Neurology, Moscow, Russia
Participants	Medical students, year not mentioned	Third-year medical students	Second-year medical students	Neurology residents	Neurology residents
Description	Online neurological localisation game (eNLG) with modified essay questions featuring simulated patients	*The* Move: Mime-based role-play training programme of neurological semiology	Neurological version of the ‘Hat Game’	weekly presentations, followed by a game show-type oral quiz, teambased	Educational board game: *Neuropoly,* pilot study
Number of participants	*n* = 76	*The Move*: *n* = 186 Standard teaching alone: *n* = 366	*n* = 107	Intervention: *n* = 17 Historical control (lectures): *n* = 20	*n* = 51
Learning assessment and outcomes	NA	Written semiology test 30 months after neurological rotation: 14% better ranking in *The Move* group (adjusted mean neurological semiology score)	Multiple Choice Questions (MCQs) before and after the game Improvement after the game: mean (±SD) score of 15.56 (±5.8) vs. 8.44 (± 4.34), *p* < 0.001	percent correct subset neurophysiology Residency Inservice Training Examination (RITE) scores: mean (±SD) score of 63.6 ± 4.12 in intervention group vs 49.4(±2.35) in control group, *p* = 0.002	Pre and post-play questionnaire: 3.2 (±1.7) vs. 7.8 (±1.6), *p* < 0.001
Satisfaction survey	93% felt the eNLG helped to better understand neurological localisation principles	NA	All students agreed that the exercise was playful	NA	Residents enjoyed the game (rate 9/10), helpful to learn neurology for 96% of participants

*NA* Not Applicable

In the future, studies should include larger samples and control groups, in the setting of multi-center collaborations, and evaluate long-term outcomes [36]. Adding a practical assessment could be considered, in order to determine whether students have acquired clinical skills.

## Conclusions

In conclusion, considering the improvement of students’ performance and positive feedback, the Neurospeed game appears to be an interesting pedagogical tool for teaching neurological semiology, as a complement to traditional lectures or other gamified teachings. These results need to be confirmed by a randomized controlled study assessing long-term memory and comparing the Neurospeed game to traditional lecturing and to other types of gamified learning such as the Neurological Hat Game.

## Supplementary Information

**Additional file 1.** List of the neurological symptoms or signs written on each card.

**Additional file 2.** Multiple Choice Questions.

**Additional file 3.** Satisfaction Questionnaire.

## Data Availability

The datasets used and analysed during the current study are available using the following links: https://docs.google.com/spreadsheets/d/1kOPJJBHub6cc_ebbdJ24wRcW4uOh4uSi/edit#gid=1320464670 https://docs.google.com/spreadsheets/d/1Sdr9p4eH1u-A5xyyEaZY-bAhjtKCrTJd/edit#gid=1857241676

## Abbreviation

MCQs: Multiple Choice Questions
