# Supplementary material for: The “Neurospeed” game: a fun tool to learn the neurological semiology
Source: BMC Med Educ. 2022 Mar 31;22:224. doi: 10.1186/s12909-022-03316-8 (PMC8970646; doi:10.1186/s12909-022-03316-8)
Supplement: Supplementary file 1 — Additional file 1. List of the neurological symptoms or signs written on each card. [file 12909_2022_3316_MOESM1_ESM.docx]

**Additional file 1. List of the neurological symptoms or signs written on each card**

|  | **English version** | **French version** |
| --- | --- | --- |
| 1. | Leri’s sign | Signe de Léri |
| 2. | Agnosia | Agnosie |
| 3. | Apraxia | Apraxie |
| 4. | Headache | Céphalée |
| 5. | Froment maneuver | Manoeuvre de Froment |
| 6. | Dischronometria | Dischronométrie |
| 7. | Osteotendinous reflexes | Réflexes ostéo-tendineux |
| 8. | Dysarthria | Dysarthrie |
| 9. | Idiomuscular reflex | Réflexe idio-musculaire |
| 10. | Vertigo | Vertige |
| 11. | Souques’ sign | Signe des cils de Souques |
| 12. | Mingazzini’s maneuver (lower limbs) | Manoeuvre de Mingazzini (membres inférieurs) |
| 13. | Akinesia | Akinésie |
| 14. | Aphasia | Aphasie |
| 15. | Hypomimia | Hypomimie |
| 16. | Disorientation | Désorientation |
| 17. | Hoffmann’s sign | Signe de Hoffmann |
| 18. | Myotonia | Myotonie |
| 19. | Phonophobia | Phonophobie |
| 20. | Cramp | Crampe |
| 21. | Nystagmus | Nystagmus |
| 22. | Fukuda’s maneuver | Manoeuvre de Fukuda |
| 23. | Patellar clonus | Clonus de la rotule |
| 24. | Ballism | Ballisme |
| 25. | Gowers’ sign | Signe de Gowers |
| 26. | Myalgia | Myalgie |
| 27. | Charles Bell’s sign | Signe de Charles Bell |
| 28. | Lead pipe rigidity | Rigidité plastique/en tuyau de plomb |
| 29. | Photophobia | Photophobie |
| 30. | Romberg’s sign | Signe de Romberg |
| 31. | Stomping gait | Marche talonnante |
| 32. | Cophosis | cophose |
| 33. | Steppage gait | Marche steppante |
| 34. | Dysmetria (Hypo/hypermetria) | Dysmétrie (Hypo/hypermétrie) |
| 35. | Tic | Tic |
| 36. | Grasping | Grasping |
| 37. | Circumduction (spastic) gait | Fauchage (marche fauchante) |
| 38. | Freezing | Freezing (enrayage cinétique) |
| 39. | « tabouret » sign (i.e., difficulty getting up from a chair) | Signe du tabouret |
| 40. | Barré’s maneuver (upper limbs) | Manoeuvre de Barré (membres supérieurs) |
| 41. | Proprioceptive ataxia | Ataxie proprioceptive |
| 42. | Confusion | Confusion |
| 43. | Hypoesthesia | Hypoesthésie |
| 44. | Asynergia | Asynergie |
| 45. | Collectionism | Collectionisme |
| 46. | Waddling gait | Marche dandinante |
| 47. | Lasègue’s sign | Signe de Lasègue |
| 48. | Brisk osteotendinous reflexes | Réflexes ostéo-tendineux vifs |
| 49. | Spasticity | Spasticité |
| 50. | Kinesthesia | Kinesthésie |
| 51. | Cerebellar ataxia | Ataxie cérébelleuse |
| 52. | Kernig’s sign | Signe de Kernig |
| 53. | Adiadocokinesia | Adiadococinésie |
| 54. | Perseveration | Persévération |
| 55. | Walking with small steps | Marche à petits pas |
| 56. | Hypopallesthesia | Hypopallesthésie |
| 57. | Hypotonia | Hypotonie |
| 58. | Babinski’s sign | Signe de Babinski |
| 59. | Dysesthesia | Dysesthésie |
| 60. | Abnormal gait | Marche anormale |
| 61. | Micrographia | Micrographie |
| 62. | Chorea | Chorée |
| 63. | Tinnitus | Acouphène |
| 64. | Ankle clonus | Trépidation épileptoïde |
| 65. | Stewart-Holmes’ maneuver | Manoeuvre de Stewart-Holmes |
| 66. | Hyposmia | Hyposmie |
| 67. | Amyotrophia | Amyotrophie |
| 68. | Lhermitte’s sign | Signe de Lhermitte |
| 69. | Ataxia | Ataxie |
| 70. | Myoclonus | Myoclonie |
| 71. | Tremor | Tremblement |
| 72. | Fasciculation | Fasciculation |
| 73. | Brudzinski’s sign | Signe de Brudzinski |
| 74. | Dystonia | Dystonie |
| 75. | Motor weakness | Déficit moteur |
| 76. | Ophtalmoplegia | Ophtalmoplégie |
| 77. | Imitation | Imitation |
| 78. | Pendular reflexes | Réflexes pendulaires |
