# Supplementary material for: The “Neurospeed” game: a fun tool to learn the neurological semiology
Source: BMC Med Educ. 2022 Mar 31;22:224. doi: 10.1186/s12909-022-03316-8 (PMC8970646; doi:10.1186/s12909-022-03316-8)
Supplement: Supplementary file 2 — Additional file 2. Multiple Choice Questions. [file 12909_2022_3316_MOESM2_ESM.docx]

**Additional file 2. Multiple Choice Questions**

**A lesion of the posterior column-medial lemniscus pathway may result in:**

1. Pyramidal syndrome

2. Ataxia

3. Positive Romberg’s sign

4. Cophosis

5. Positive Lhermitte’s sign

**In the pyramidal syndrome, there is/are:**

1. Clonus of the patella

2. Babinski’s sign

3. hypoesthesia

4. Cophosis

5. Circumduction (spastic) gait

**Amongst the following signs, which one relates to an impairment of instrumental functions:**

1. Akinesia

2. Apraxia

3. Agnosia

4. Asterixis

5. Aphasia

**Amongst the following symptoms, which one designates a movement disorder:**

1. Tic

2. Ballism

3. Chorea

4. Myoclonus

5. Dystonia

**Peripheral neuropathy can manifest with:**

1. Myalgia

2. Fasciculations

3. Waddling gait

4. Frontal syndrome

5. Diminished deep tendon reflexes

**An affection of the neuromuscular junction may lead to:**

1. Myotonia

2. Positive Hoffman’s sign

3. Ptosis

4. Ophthalmoplegia

5. Ataxia

**In the vestibular syndrome, one can observe:**

1. Hyposmia

2. Epilepsy

3. Positive Froment’s maneuver

4. Vertigo

5. Positive Fukuda stepping test

**What are the different kinds of abnormal gait:**

1. Dancing gait

2. Sensory ataxia

3. Steppage gait

4. Waddling gait

5. Jumping gait

**A nystagmus may be observed in:**

1. Vestibular syndrome

2. Peripheral nerve syndrome

3. Dementia

4. Cerebellar syndrome

5. Myogenic syndrome

**In a frontal syndrome, one can observe:**

1. Disinhibited behavior

2. Apathy

3. Altered attention

4. Perseverations

5. Collectionism

**In a cerebellar syndrome, one can find:**

1. Tremor

2. Adiadococinesia

3. Dyschronometry

4. Pendular deep tendon reflexes

5. Asynergia

**Select the correct sentences:**

1. Deep Tendon Reflexes (DTR) are brisk in the cerebellar syndrome

2. DTRs are brisk in the pyramidal syndrome

3. DTRs are diminished in the myasthenic syndrome

4. DTRs are diminished in the peripheral nerve syndrome

5. DTRs are normal in the vestibular syndrome

**In an advanced myogenic syndrome, one can observe:**

1. Abolition of DTR

2. Amyotrophia

3. Increased idiomuscular contractions

4. Steppage gait

5. Fasciculations

**Which of the following are signs of the pyramidal syndrome:**

1. Romberg’s sign

2. Babinski's sign

3. Brudzinski's sign

4. Hoffmann’s sign

5. Froment’s sign

**Parkinsonian akinesia can manifest with:**

1. Freezing

2. Dystonia

3. Steppage gait

4. Hypophonia

5. Hypersalivation

**Hypotonia is found in:**

1. Parkinsonian syndrome

2. Vestibular syndrome

3. Chorea

4. Pyramidal syndrome

5. Cerebellar syndrome

**Ataxia can be:**

1. Cerebellar ataxia

2. Pyramidal ataxia

3. Nerve trunk ataxia

4. Vestibular ataxia

5. Epileptic ataxia

**Dysarthria can be:**

1. Paretic dysarthria

2. Parkinsonian dysarthria

3. Vestibular dysarthria

4. Frontal dysarthria

5. Ataxic dysarthria

**In a meningeal syndrome, one can find:**

1. Kernig’s sign

2. Souques’ sign

3. Phonophobia

4. Confusion

5. Neck stiffness

**In an L5 root lesion, one can observe:**

1. Lasègue’s sign

2. Léri’s sign

3. Abolition of the patellar jerk reflex

4. Gowers’ sign

5. Sensory disorders of the lower limb
