# Supplementary material for: The “Neurospeed” game: a fun tool to learn the neurological semiology
Source: BMC Med Educ. 2022 Mar 31;22:224. doi: 10.1186/s12909-022-03316-8 (PMC8970646; doi:10.1186/s12909-022-03316-8)
Supplement: Supplementary file 3 — Additional file 3. Satisfaction Questionnaire. [file 12909_2022_3316_MOESM3_ESM.docx]

**Additional file 3. Satisfaction Questionnaire**

The Neurospeed game is playful:

1. Strongly agree

2. Agree

3. Neutral

4. Disagree

5. Strongly disagree

The Neurospeed game is a stimulating game :

1. Strongly agree

2. Agree

3. Neutral

4. Disagree

5. Strongly disagree

The Neurospeed game helped better understand neurological semiology :

1. Strongly agree

2. Agree

3. Neutral

4. Disagree

5. Strongly disagree

The Neurospeed game helped better remember neurological semiology :

1. Strongly agree

2. Agree

3. Neutral

4. Disagree

5. Strongly disagree

The Neurospeed game was useful for reviewing the upcoming exam

1. Strongly agree

2. Agree

3. Neutral

4. Disagree

5. Strongly disagree

The Neurospeed game increased motivation to learn neurological semiology

1. Strongly agree

2. Agree

3. Neutral

4. Disagree

5. Strongly disagree

Terms used for the Neurospeed game were appropriate

1. Strongly agree

2. Agree

3. Neutral

4. Disagree

5. Strongly disagree

The Neurospeed game should be repeated in the future

1. Strongly agree

2. Agree

3. Neutral

4. Disagree

5. Strongly disagree

The Neurospeed game should be extended to other medical specialties

1. Strongly agree

2. Agree

3. Neutral

4. Disagree

5. Strongly disagree
